# Their True Relationship

**Published:** 1919-11-08

**Authors:** 


### THE MINISTRY OF HEALTH AND THE NURSE.

### Their True Relationship.

A Hospital correspondent suggests that I hold a brief for the Ministry of Health. I had not really thought about the matter before, but, on reflection, I am disposed to plead guilty.

Have you ever after a long and weary journey found your hotel porter in uniform on the platform? If so, you probably never heard his name, or noticed if he was dark or fair. All the same, you were not a little pleased to find him awaiting your arrival. You appropriated him, handed over all your possessions, and thanked God for His mercies. To-morrow and the day after you saw him again. He knew exactly what you wanted done and did it. By the time you left you had quite a kindly feeling for the man. And if ever you have occasion to travel this way again you will again look out for him. He will probably be quite a different individual. But he will hold the same office, wear the same uniform, and have the same wonderful way of anticipating your wishes. It is, if you will analyse it, not the man that you appreciate, but the office. The hotel provides you with a highly-trained, liveried servant during your stay. Very few visitors there are to this hostelry who do not hold a metaphorical brief for him.

### Suspicion and Distrust.

The Minister of Health corresponds with the hotel servant. Not any of the visitors can claim him as theirs exclusively. It does not matter whether his name is Smith or Jones. He has an extremely important and difficult task to grapple with, a task given to him by the State, and therefore by me as much as by anybody else. He is my servant, and I suggest that nothing is more unjust than to treat a servant with "suspicion and distrust" without very well-established evidence. A lady of my acquaintance the other day lost her pet thimble. She immediately called the little maid whom she employs and charged her with being a thief, but within an hour she had to offer a humble and shamefaced apology.

Now, this correspondent who preaches suspicion and distrust of the Ministry of Health appears to me to be emulating the lady with the thimble. There is not a particle of evidence cited that this Department of the State has betrayed any trust or acted in any unworthy manner. I am told that the Cottage Benefit Nursing Association "are advertising in the daily papers for women who, on completion of one year's training, are assigned the status of a nurse, and are expected to combine in their work the duties of a professional woman and a home help, to the detriment of both." The Minister of Health is blamed for this because of the State Registration Bill not being yet forthcoming. I do not know what kind of Bill "L. E. G." expects, but neither the College Bill nor its rival contained any clause which would prevent the Cottage Benefit Nursing Association from appointing nurses without any training at all. Will people never understand that no Registration Act ever will or ever can confer a monopoly of nursing for gain on trained nurses? I have stated and re-stated this obvious and elementary fact for the benefit of Registration enthusiasts of all schools. They never argue back, but they go on all the time assuming that when the Act passes the so-called "nurse" will be put out of business. This has occurred so often that my mind is not free from "suspicion and distrust" of those leaders who insist on ignoring the vital interests of the profession, and alluring them by devious ways into the wilderness.

To come back to the health visitor. I have carefully studied the literature issued by the Ministry of Health, and I am convinced that a hospital training is not sufficient for turning out an effectual health visitor. The fact is obvious. Let us try to be judicial. In the literature referred to the Ministry literally beg for the services of trained nurses. They beg for the co-operation of hospitals and the leaders of the profession. They set out their curriculum, and they go out of their way to make clear that the rules are not hard and fast, and that the Ministry are always open to suggestion and willing to co-operate in order to facilitate the nurse in fitting herself for her new employment. The answer is "suspicion and distrust." And in the meantime rank outsiders are capturing the prizes, and women's colleges are being subsidised to give whoever comes along the necessary training. I must confess that I am utterly unable to understand the attitude. If it is honest it is stupid. If it is not honest it is wicked. And, looking backward, it appears to me that every conceivable will-o'-the-wisp is made the subject of a faction fight, and attention thus deliberately taken away from the only thing that is worth considering outside the day's duty—namely, better pay, a wider outlook, and greater personal freedom. One party sets up a State Registration stunt, another composes a bogus Charter of Liberty, a third deliberately derides the Nation's Tribute as an insult.

So on and so forth. The Ministry of Health has opened the widest door that has yet appeared possible, and invited nurses to prepare and enter into possession. What is the answer? There is none save a vague and unsubstantiated charge of *mala fides*. The offer is treated with "distrust and suspicion."

### A Call for Frank Expression.

If I am any judge of the portents this charge will be found to possess some of the characteristics of a boomerang. "L. E. G." mentions the College. What is it doing about this Health Ministry programme? It is, amongst other things, an educational institution. I have been patiently waiting week by week to hear of a plan of campaign calculated to bring hospitals into alignment with post-war schemes of health reform and reconstruction. Every week's delay means the outsider working her way into the legitimate domain of the trained nurse and the certified midwife. If the College is to fulfil its destiny there is no time to lose. I have been hearing rumblings of discontent and anger from amongst the rank and file that their claims for recognition are being neglected. The distrustful and suspicious attitude will not help. If there are grounds for such, let it always be borne in mind that it is a public Department of State, and not the private enterprise of an individual or a party.

Frank and free expression of opinion is urgently called for at this juncture. If there are causes for complaint set them out on the printed page. The Nursing Press is strong enough to set its house in order.

IERNE.

